# Molecular regulation and therapeutic implications of cell death in pulmonary hypertension

**DOI:** 10.1038/s41420-023-01535-6

**Published:** 2023-07-12

**Authors:** Enze Wang, Sijing Zhou, Daxiong Zeng, Ran Wang

**Affiliations:** 1 Department of respiratory and critical care medicine, the first affiliated hospital of Anhui medical university, Hefei, 230022 China; 2 Department of Occupational Disease, Hefei third clinical college of Anhui Medical University, Hefei, 230022 China; 3 Department of pulmonary and critical care medicine, Dushu Lake Hospital Affiliated to Soochow University, Medical Center of Soochow University, Suzhou, 215006 China

**Keywords:** Cell death, Cardiovascular diseases

## Abstract

Pulmonary hypertension (PH) is a clinical and pathophysiological syndrome caused by changes in pulmonary vascular structure or function that results in increased pulmonary vascular resistance and pulmonary arterial pressure, and it is characterized by pulmonary endothelial dysfunction, pulmonary artery media thickening, pulmonary vascular remodeling, and right ventricular hypertrophy, all of which are driven by an imbalance between the growth and death of pulmonary vascular cells. Programmed cell death (PCD), different from cell necrosis, is an active cellular death mechanism that is activated in response to both internal and external factors and is precisely regulated by cells. More than a dozen PCD modes have been identified, among which apoptosis, autophagy, pyroptosis, ferroptosis, necroptosis, and cuproptosis have been proven to be involved in the pathophysiology of PH to varying degrees. This article provides a summary of the regulatory patterns of different PCD modes and their potential effects on PH. Additionally, it describes the current understanding of this complex and interconnected process and analyzes the therapeutic potential of targeting specific PCD modes as molecular targets.

## Facts

- The transition from pro-apoptosis to anti-apoptosis of pulmonary vascular cells accompanies the development of PH, which needs to clarify the proper intervention time of therapy.
- Necroptosis regulates PH mainly through non-classical regulatory pathways, which may be a protective mechanism after apoptosis is inhibited.
- PH is always accompanied by increased autophagy; however, it plays opposite roles at different stages of the disease.
- Although it is contrary to cell death, pyroptosis can promote pulmonary vascular cell proliferation, induce vascular remodeling, and exacerbate PH. The exact molecular mechanism remains to be investigated.
- There are imbalances in both iron homeostasis and copper homeostasis in PH. The activation and inhibition of ferroptosis seem to exist simultaneously in pulmonary vascular cells. Cuproptosis may be a promising therapeutic target for PH.

## Introduction

The prevalence of pulmonary hypertension (PH) is estimated to be ~1% of the global population and may peak at >10% in people older than 65 years of age [1]. With the release of the latest 2022 ESC/ERS Guidelines for the diagnosis and treatment of PH, the threshold for PH diagnosis has been lowered from 25 mmHg to 20 mmHg [2], which means that more patients with potential PH will be identified, and receive attention. Pulmonary vascular endothelial dysfunction, smooth muscle layer thickening, extracellular matrix deposition, and pulmonary vascular remodeling are typical pathological features of PH and are regulated by various molecular signaling pathways [3, 4].

Cell death is the final destination of all biological cells and is necessary to maintain normal homeostasis in organisms. Unlike cell necrosis, a passive process of cell death, programmed cell death (PCD) is an active cell death mode initiated by gene regulation when cells are stimulated by internal or external environmental factors [5, 6]. Depending on the molecular mechanism, PCD can be divided into various types: apoptosis, autophagy, pyroptosis, ferroptosis, necroptosis, and cuproptosis [7]. Recent studies have shown that abnormal PCD can result in tumors, immune disorders, inflammation, and other diseases [8, 9]. In patients with PH, PCD plays a key role in increasing both pulmonary vascular pressure and remodeling [10]. In this paper, we summarize the regulatory signaling pathways for a few types of PCD to explain the complex and interlaced processes and review their role in the progression of PH disease.

## Apoptosis and necroptosis

### Process of apoptosis

**Fig. 1:**
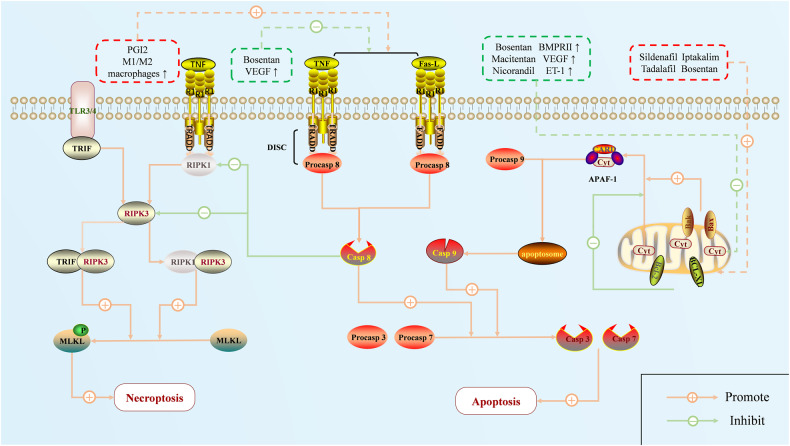
The processes and regulation of apoptosis and necroptosis in PH. Both the intrinsic and external pathways of apoptosis are involved in the pathogenesis of pulmonary hypertension. The activation or inhibition of apoptosis is required in different scenarios to slow the progression of pulmonary hypertension, while necroptosis is more of a protective mechanism after apoptosis is inhibited. Red represents the enabler, and green represents the inhibitor.

Apoptosis is the earliest discovered PCD, and its regulation patterns have been widely studied. Current research on this topic is focused on the intrinsic and external pathways (Fig. 1) [11, 12]. Apoptosis-related regulatory molecules are activated when the cell receives an internal or external apoptotic signal. The members of the caspase protein family (a key protein in apoptosis) are activated, resulting in a protease cascade amplification reaction process in which apoptotic signals are irreversibly amplified [12, 13]. During this period, morphological changes, such as chromatin condensation, nuclear fragmentation, cell contraction, and the formation of apoptotic bodies, can be observed in cells [14]. Finally, when macrophages recognize phosphatidylserine (PtdSer) exposed on the cell surface, apoptotic bodies are phagocytosed by macrophages [15].

#### External pathway of apoptosis

Death receptors trigger the external apoptotic pathway. Death signals from the external environment or the body system are delivered to the cell via death receptors that activate death ligands. Various apoptosis-related combinations have been identified, including TNF/TNFR1/2, FasL/FasR, and TRAIL/DR4,5 [16]. On most cell surfaces, when TNF binds to TNFR or FasL binds to FasR, the TNF receptor-associated death domain (TRADD) or FAS-associated death domain (FADD) is recruited to the cytoplasm to form a complex, which in turn binds procaspase-8 [12, 17]. The receptor, ligand, and procaspase-8 together constitute the death-inducing signaling complex (DISC) that promotes the activation of caspase-8 [18]. An apoptotic cascade reaction is induced to initiate cell death by the proteolytic cleavage of procaspase-3 and procaspase-7.

#### Intrinsic pathway of apoptosis

The intrinsic pathway of apoptosis, also known as the mitochondrial pathway, is usually induced by endogenous apoptotic signals such as hypoxia, DNA damage, and a lack of growth factors [12]. The mitochondrial outer membrane permeabilization increases and cytochrome C is released into the cytoplasm [19]. Cytochrome C is sensed by the apoptotic protease activator 1 (APAF-1). Next, procaspase-9 is recruited through the caspase activation and recruitment domain (CARD) of APAF-1 [16]. Cytochrome C, APAF-1, and procaspase-9 form the apoptotic body complex, activating caspase-3 and caspase-7 to perform the executioner function. The B-cell lymphoma 2 (BCL-2) family proteins are key regulatory proteins in the intrinsic pathways that affect mitochondrial membrane permeability [20, 21]. Members of this family include pro-apoptotic effector proteins (i.e., BAX, BAK, and BOK), which increase membrane permeability, and anti-apoptotic effector proteins (i.e., BCL-2, BCL-XL, and MCL-1), which prevent membrane permeability [22]. Two types of proteins are involved in homeostasis to regulate the cell response to apoptotic signals [23]. Apoptosis occurs as soon as this equilibrium is disturbed.

### Altered apoptotic state in PH

The typical pathological changes of PH are the hyperplasia of the smooth muscle layer and intima of the pulmonary artery, and this indicates that the abnormal state of pulmonary arterial endothelial cells (PAECs) and pulmonary artery smooth muscle cells (PASMCs) play a key role in PH. In fact, in hypoxic pulmonary hypertension (HPH), the apoptosis of PAECs is a potential trigger in the early stages of PH, and PAECs often undergo a transition from the pro-apoptotic to anti-apoptotic phenotype with disease progression [24]. For example, in the early stage of the monocrotaline (MCT)-induced HPH rat model, polarized M1 macrophages can be observed infiltrating the perivascular and alveolar spaces, accelerating the apoptosis of PAECs. In contrast, in the vascular remodeling phase, pro-proliferative M2 macrophages are dominant [25, 26]. In addition, in patients at the early stage of PH, the expression of anti-apoptotic markers (e.g., Bal-2 and eNOS) is usually lower in PAECs, and there is almost no intimal hyperplasia in the lung tissue [27]. A classical hypothesis suggests that PAECs are damaged by various environmental factors, such as chronic hypoxia or shear stress, leading to massive apoptosis. While repair mechanisms such as proliferation and migration of neighboring PAECs or homing of circulating endothelial progenitor cells maintain pulmonary circulation integrity. Long-term PAEC loss will selectively preserve PAECs that are tolerant to apoptosis and overproliferate, eventually leading to irreversible intimal hyperplasia and plexiform lesions [24, 26]. However, PASMCs were always in an active anti-apoptotic state during the progression of PH. Our previous study observed abnormal activation of anti-apoptotic signaling in PASMCs during the early stage of hypoxia [28].

Several important factors and pathways regulate this process. The abnormal expression of pathogenic genes (e.g., BMPR2, CAV1) within the pulmonary vascular cells will produce different degrees of apoptotic effects on PASMCs and PAECs [29, 30]. Although the exact mechanism is still unclear, a mutation in bone morphogenetic protein type II receptors (BMPR2) inhibits the expression of the anti-apoptotic protein Bcl-XL and enhances apoptosis susceptibility in PAECs while promoting apoptosis tolerance by increasing the BcL-XL/BcL-Xs ratio through alternative splicing of the Bcl-x pre-mRNA in PASMCs [30, 31]. In the cellular microenvironment, changes in the levels of various cytokines accompany the progression of PH. Vascular endothelial growth factor (VEGF) is a star molecule that regulates the mitosis and growth state of endothelial cells (ECs) and can induce apoptosis tolerance in ECs through the external pathway of Fas signaling and the intrinsic pathway of the Bcl-2 protein [32, 33]. In various PH models, such as hypoxia, fibrosis, thromboembolism, and hypothermia, VEGF-decreased expression levels are closely related to EC apoptosis at the early stage of PH [24, 34–36]. However, in the advanced stage, anti-apoptotic PAECs and PASMCs interact with each other by producing excessive growth factors (e.g., TGF-β, FGF2, ET-1) and exosomes through autocrine and paracrine pathways, which further aggravates the proliferation of both [30, 37, 38]. Other factors that produce an apoptotic imbalance in pulmonary vascular cells include elevated plasma NO [39], the inhibition of potassium channels in the membrane by external stimuli [40], and the accumulation of intracellular ROS, which upsets the balance of ROS and leads to oxidative stress [41, 42].

### Apoptosis and the treatment of PH

Apoptosis is one of the most critical mechanisms by which PH occurs; as a result, researchers are paying more attention to assessing drug efficacy in terms of apoptosis. Although there is still a lack of research on the specific mechanism of PH-targeting drugs regulating pulmonary vascular cell apoptosis, it is clear that among the three targeted therapy pathways, the nitric oxide pathway (e.g., phosphodiesterase 5 inhibitors) and prostacyclin pathway (e.g., prostacyclin receptor agonists) can promote the apoptosis of both PASMCs and PAECs to reverse pulmonary vessel wall thickening through the mitochondrial pathway and extrinsic pathway, respectively [43–46]. However, in the endothelin pathway, endothelin receptor antagonists (ERA) inhibit the proliferative effect of ET-1 on PASMCs; however, they inhibit the apoptosis of PAECs by activating the Bcl-2/caspase-3 pathway [47], and this is reflected in the fact that the expression of VEGF is inhibited in the lung tissue and increased in the pulmonary blood vessels with the treatment of dual ERA [48]. Because of the different distributions of endothelin receptors (ET) in PASMCs and PAECs (ET-A and ET-B in PASMCs; only ET-B in PAECs), it appears that non-selective antagonists can inhibit the pro-proliferative ET-A in PASMCs. However, this effect is greatly attenuated in PAECs [49]. Similar pharmacological effects have been observed for other drugs that can affect the PH-related apoptosis process. For example, the ATP-sensitive potassium channel opener, iptakalim, can promote PASMC apoptosis in patients with HPH through mitochondrial pathways [50]. However, nicorandil, which has similar pharmacological effects, can activate p38 MAPK and upregulate eNOS to protect PAECs from apoptosis [51]. Considering the apoptotic manifestations of PH at different stages, heritable pulmonary arterial hypertension is typically found in the advanced stage and requires pro-apoptotic drugs to relieve the thickening of the pulmonary vascular membrane. However, in the early stages of traumatic pulmonary arterial hypertension, anti-apoptotic drugs are required to prevent the occurrence of apoptotic waves in PAECs and protect pulmonary circulation. Additionally, this explains, to a certain extent, why it is often difficult for a few IPAH-targeting drugs to achieve a good prognosis in the third PH group [2]. Most current studies remain in the experimental animal stage. The underlying molecular biological mechanisms still need to be explored, and further clinical experimental evidence is needed.

### Necroptosis and PH

The activation signals of necroptosis are the same as those of apoptosis. However, necroptosis is a regulated inflammatory death process that manifests as membrane perforation, cytoplasmic disintegration, and content release (Fig. 1) [52]. The activated death domain induces RIPK1, which recruits RIPK3 to form necrosomes. These necrosomes phosphorylate MLKL and perform necroptosis [53]. On the one hand, MLKL can change the permeability of ion channels in the membrane and cause cell rupture. On the other hand, MLKL causes membrane translocation by binding to phosphatidylinositol, directly destroying the integrity of the cell membrane [54, 55]. In addition, a few receptors, such as TLR3/4, can bypass RIPK1 and directly activate RIPK3 phosphorylation to induce the non-classical regulatory pathway [56]. However, RIPK1 and RIPK3 can be cleaved and inactivated by caspase-8, implying that cells can initiate necroptosis only when the extrinsic apoptotic pathway is inhibited [57].

To a certain extent, researchers tend to think that necroptosis is a fail-safe mechanism in cells following apoptosis inhibition [58]. Therefore, most current studies suggest that, due to the anti-apoptotic properties of pulmonary vascular cells in PH, necroptosis is correspondingly activated and primarily regulated by the non-classical pathway. For instance, bioinformatics analysis has revealed activation of the TLR and NLR pathways and upregulation of RIPK3 and MLKL mRNA, and downregulation of RIPK1 mRNA in the transcriptome of the lung tissue of HPH rats [59]. In PASMCs, the damage-related molecular pattern HMGB1 activates the downstream TLR4 pathway and causes an inflammatory response [60]. Recent studies have confirmed the activation of phosphorylated RIPK3 and phosphorylated MLKL and the significant inhibition of activated caspase-8 in the right ventricle of HPH rats, suggesting that necroptosis mediates right ventricular injury in PH [61]. However, whether this passively activated pattern of PCD affects PH progression is a question that requires further investigation.

## Autophagy

**Fig. 2:**
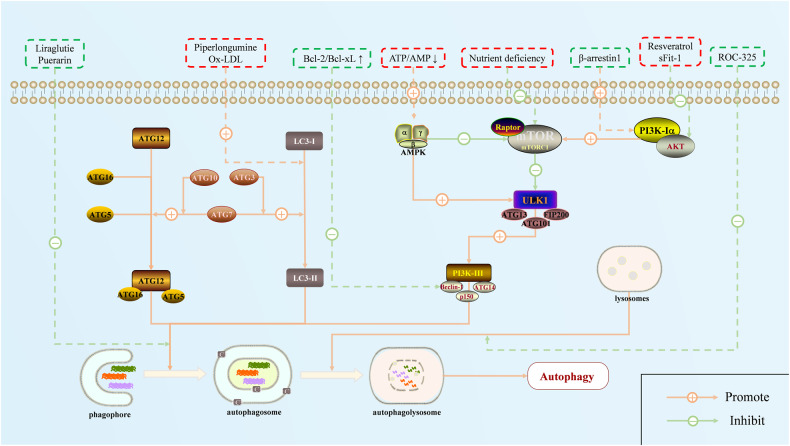
The process and regulation of autophagy in PH. Pulmonary hypertension can be alleviated by activating or inhibiting the autophagy of pulmonary vascular cells. Studies on the relevant mechanisms involve all aspects of the autophagy process, which is mainly regulated through the mTOR pathway. Red represents the enabler, and green represents the inhibitor.

Autophagy is an intracellular catabolic process in which cell solutes and/or certain contents enter lysosomes for degradation under the regulation of the autophagy-related gene (ATG) protein family, thereby fulfilling the metabolic needs of cells and the renewal of a few organelles (Fig. 2) [62]. Autophagy can be divided into three types according to the different inclusions and transport modes: macroautophagy, microautophagy, and chaperone-mediated autophagy [63]. The formation of a unique autophagosome characterizes macroautophagy, which is the most widely studied type of autophagy [64]. The term autophagy used in this review refers to macroautophagy.

Autophagy occurs when cells are exposed to various external stimuli, such as nutrient deficiency, growth factor deficiency, and hypoxic and cytotoxic substances [65]. The initiation of autophagy depends on the activation of the core signal ULK1 complex (a serine/threonine protein kinase complex composed of ULK1, ATG13, FIP200, and ATG101), which activates the downstream class III phosphatidylinositol 3 kinase (PI3K) complex and promotes the formation of phagophores [66]. Phagophores are double-layered membrane structures surrounding the damaged cytoplasm and organelles in the inner membrane of cells [67]. Subsequently, the Atg5-Atg12-Atg16L complex and microtubule-associated protein light chain 3-II (LC3-II, also known as ATG8) are formed and fused by phagophores to form autophagosomes [66, 68]. Autophagosomes capture proteins, organelles, and other substances that need to be degraded or eliminated and combine with lysosomes to form autophagolysosomes. Finally, autophagosomes and their contents are hydrolyzed into macromolecular substances, such as amino acids, in the autophagolysosome and released back to the cytoplasm for reuse [63, 69]. These processes are regulated by more than 40 ATGs and multiple signaling pathways and proteins [70], the most notable of which are the mammalian target of rapamycin (mTOR) pathway, the AMP-activated protein kinase (AMPK) pathway, and the proteins Beclin-1 and LC3-II.

### Key regulatory signals for autophagy

#### PI3K/AKT/mTOR signaling pathway

The PI3K/AKT/mTOR pathway is widely involved in cell survival and growth under physiological conditions [71]. After stimulation with various cytokines, PI3K is activated further to recruit the downstream serine/threonine kinase AKT to phosphorylate mTOR [72]. mTOR is composed of mTORC1 and mTORC2, with mTORC1 mainly sensing the nutritional status of cells [73]. Under conditions of cellular nutrient deficiency, a decrease in mTORC1 inhibits the phosphorylation of the ULK1 complex and subsequently initiates autophagy [73, 74].

#### AMPK/ULK1 signaling pathway

The AMPK/ULK1 pathway acts during the initiation of autophagy. Unlike mTORC1, AMPK is an activator of autophagy [75]. Under the stress conditions of nutrient deficiency, AMPK senses the intracellular ATP/AMP ratio and is activated by the phosphorylation of upstream serine/threonine kinases [76]. In contrast, activated AMPK phosphorylates mTORC1, facilitating its dissociation from the ULK1 complex, and AMPK phosphorylates the ATG13 and FIP200 groups of the ULK1 complex, thereby activating this complex to initiate autophagy [77–79].

#### Beclin-1 and LC3-II

The class III PI3K complex, consisting of VPS34, Beclin-1 (ATG6), ATG14L, and p150 (VPS15), plays a major role in the nucleation of autophagosomes [66, 80]. Beclin-1 is a core protein that regulates the formation and membrane transport of this complex, which is regulated by the ULK1 complex upstream [81]. Beclin-1 activation is regulated by BCL-2/Bcl-xL, indicating a certain degree of crosstalk between autophagy and apoptosis [82]. The ATG14L group recruits LC3-II to the activated PI3K complex, which is mainly used to maintain the stability of the autophagosome membrane. Because LC3-II stably accumulates in cells, it is usually considered a good indicator for evaluating the degree of autophagy [83].

### Different roles of autophagy in PH

During the progression of PH, pulmonary vascular cells are induced into autophagy by long-term external stimuli, such as hypoxia, oxidative stress, mitochondrial dysfunction, and chronic shear stress [84–86]. Inhibition of the AMPK/mTOR signaling pathway, activated markers related to autophagy (such as the ULK1 complex, Beclin-1, and LC3-II), an abnormal increase in autophagosomes and autophagolysosomes were detected in both PASMCs and PAECs when various types of PH models were constructed [84, 87, 88]. This phenomenon is not difficult to explain. As a protective mechanism, autophagy maintains the survival of pulmonary vascular cells by timely eliminating damaged cellular organs. However, the results obtained from this dynamic mechanism for maintaining intracellular homeostasis are often subject to large uncertainties, especially in pathological conditions. In an MCT-induced PH rat model, activation of autophagy aggravates vascular endothelial dysfunction and promotes angiogenesis [89, 90]. In a hypoxia-induced PH mouse model, inhibition of autophagy by the overexpression of mTOR reverses pulmonary artery and right ventricular hypertrophy [91]. However, there is also a certain amount of evidence to support the protective effect of activated autophagy on PH. For example, although ox-LDL treatment significantly increases the autophagic flux (LC3-II/LC3-I and Beclin-1) in vascular ECs, inhibition of the PI3K/AKT/mTOR pathway and activation of autophagy by sFlt-1 effectively alleviate ox-LDL-induced apoptosis and cell damage [92].

Whether autophagy promotes or relieves PH mainly depends on the induction rate and timing of autophagy and the degree and duration of the stimulatory signals [84, 93]. Similar to apoptosis, we suggest that the enhanced autophagy that occurs in PAECs during the early stages of PH is insufficient to compensate for the impairment in response to environmental factors when environmental stress persists. As cells gradually tolerate environmental stimuli and proliferative cell growth, enhanced autophagy under the stimulus of environmental factors degrades the regulatory signals that inhibit proliferation and provide a large number of nutrients for cell reproduction, thereby exacerbating disease progression. A study of HIV-associated PH proved that the independent activation of apoptosis and autophagy could be observed in the early stages of PAECs treated with morphine and HIV-Tat, and this includes an increase in autophagosome and autophagy protein levels, with the enhanced autophagy protecting PAECs from M + T-related apoptotic stimulation [94]. However, under long-term drug therapy, persistent autophagy suppresses apoptosis in PAECs by reducing cellular ROS and inhibiting oxidative stress. The specific mechanisms by which autophagy affects PH progression and the role of autophagy in different phases of PH are important directions for future research.

### Autophagy and the treatment of PH

The potential value of regulating autophagy in alleviating PH has been recognized. However, there is still a lack of research on whether PH-targeted drugs regulate autophagy in pulmonary vascular cells. Most studies have focused on the potential therapeutic value of drugs targeting the autophagy signaling pathway in PH, specifically the mTOR pathway. However, as the potential effects of activated autophagy at different stages of PH have not been clearly defined, the existing studies describe two distinct perspectives that seem to be independent of the method used to construct the PH model and the type of pulmonary vascular cells. In PAECs, β-arrestin1 inhibits autophagy and promotes the apoptosis of hypoxia-induced PAECs by activating the AKT/mTOR signaling pathway [95]. In contrast, resveratrol alleviates PAEC dysfunction in CTEPH rats by enhancing autophagy [96]. Similarly, puerarin improves the proliferation of hypoxia-induced PASMCs by inhibiting autophagy [97]. In contrast, piperlongumine increases the autophagic flux of PASMCs and alleviates pulmonary artery wall thickness, pulmonary arteriolar muscularization, and right ventricular hypertrophy in rats in a hypoxic environment [98]. Therefore, before using autophagy modulators, we should carefully consider whether our interventions are consistent with the pathological course of PH. Further research is necessary to confirm the optimal timing of autophagy interventions.

In addition to the mTOR pathway, other key autophagy processes have been identified as potential therapeutic targets for PH. Liraglutide, a glucagon-like peptide-1 receptor agonist, can inhibit the formation of autophagosomes and alleviate PASMC proliferation and migration by reducing the expression of Atg5, Atg7, and LC3B-II [99]. During the lysis of autophagosomes, ROC-325 can inhibit the formation of autophagolysosomes by increasing lysosomal membrane permeability and lysosomal deacidification, and it can significantly reduce the autophagic flux in PAECs and activate the nitric oxide signaling pathway, which in turn improves right ventricular dysfunction and pulmonary vascular remodeling in hypoxic PH rats [100]. While clinical data on the efficacy of autophagy-targeting drugs is still lacking, these novel drugs have broadened the scope of PH treatment.

## Pyroptosis

**Fig. 3:**
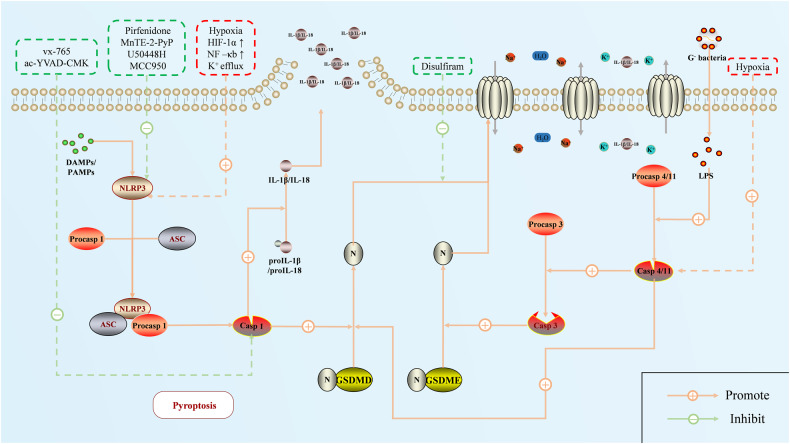
The process and regulation of pyroptosis in PH. The process of pyroptosis in pulmonary hypertension includes classical, non-classical, and caspase-3/GSDME pathways. Currently, research mainly aims to inhibit inflammasome (NLRP3) signaling to reduce pyroptosis, thus slowing the progression of pulmonary hypertension. Red represents the enabler, and green represents the inhibitor.

In recent years, pyroptosis, a novel form of PCD, has gradually become a research hotspot and has been shown to play a role in the immune, nervous, and cardiovascular systems. Microscopically, under the stimulation of inflammatory cytokines, the activation of caspases leads to the N-terminal oligomerization of gasdermins (Fig. 3). The cell membrane is then cleaved, and non-selective pores with a diameter of 1–2 μm are formed, allowing the free passage of IL-1β/IL-18 and caspase-1. Simultaneously, water can enter the cell through these small pores, causing swelling, osmosis, and plasma membrane rupture. The inflammatory response becomes more intense with the release of IL-1β and IL-18 [101, 102]. The current view is that the gasdermin family of proteins mainly mediates pyroptosis and requires the activation of inflammatory caspases or granzymes, which cleave gasdermins, to have a biological effect [103]. Depending on whether or not caspase-1 is involved, the regulatory pathway of pyroptosis is normally divided into the classical and non-classical inflammasome pathways [104].

### Regulatory pathways of pyroptosis

#### Classical regulatory pathways

The inflammasome usually initiates the classical regulatory pathway for pyroptosis. When cells are exposed to dangerous stimuli, pattern recognition receptors (such as NOD-like receptors and Toll-like receptors) are activated by recognizing pathogen-associated molecular patterns (PAMPs) and risk-associated molecular patterns (DAMPs) in the cytoplasm. They then recruit procaspase-1 and apoptosis-associated speck-like proteins containing CARD (ASC) to form inflammasomes [104–106]. Inflammasomes activate caspase-1 and cleave the cell membrane via GSDMD, leading to pyroptosis. Caspase-1 cuts proIL-1β and proIL-18 to release many inflammatory mediators [107–109].

#### Non-classical regulatory pathways

The non-classical inflammasome pathway for pyroptosis mainly relies on human caspase-4/5 or murine caspase-11. Currently, research on this pathway mainly focuses on infectious diseases [110, 111]. Typically, these caspase precursors are activated by LPS and cleave GSDMD, forming cell membrane pores. These processes do not require inflammasomes [112]. Although these caspases do not directly activate IL-1β and IL-18, they can induce potassium efflux through non-selective pores on the membrane, thereby activating the NLRP3 inflammasome and up-regulating caspase-1, which indirectly activates the classical regulatory pathway of pyroptosis [112, 113].

#### Other Approaches

Recent studies have shown that pyroptosis can be induced under certain circumstances. For example, cytotoxic lymphocytes can release granzyme A (GzmA) to cut GSDMB, mediating the occurrence of pyroptosis in target cells [114]. The nuclear translocation of PD-L1 in tumor cells promotes the expression of GSDMC, which is cleaved by caspase-8 and promotes the transformation from apoptosis to pyroptosis [115]. In addition, similar pathways include caspase-8/GSDMD [116] and granzyme B (GzmB)/GSDME [117]. Caspase-3 is usually considered an apoptosis regulator; however, studies have shown that activated caspase-3 cleaves GSDME under the stimulation of a few drugs [118]. Whether caspase-3 causes pyroptosis or apoptosis depends on the level of GSDME, which is highly expressed and promotes the caspase-3-induced pyroptosis pathway.

### Activated pyroptosis pathways promote PH

The study of pyroptosis in PH is still limited. Most researchers believe that pyroptosis is activated in PH and promotes the proliferation of pulmonary vascular cells, increased pulmonary artery pressure, pulmonary vascular remodeling, and right ventricular hypertrophy [119, 120]. It is puzzling why this inflammatory death program occurs in a large number of pulmonary vascular cells, yet it results in an increase in the total number of cells and a thickening of the pulmonary artery wall. One view is that inflammatory mediators released by pyroptosis, such as IL-1β and IL-18, promote the activation of pro-proliferation signaling pathways in the surrounding cells [120]. However, this does not explain why pyroptosis continues to be active in the course of PH, both in PASMCs and PAECs [121]. Our lack of knowledge regarding the mechanisms underlying all of this is a pressing issue that needs to be addressed.

Multiple pyroptosis pathways have been demonstrated to be involved in regulating PH. The NLRP3 inflammasome complex, which is composed of the immune receptor NLRP3 protein, ASC, and caspase-1, plays a key role in the classical pathway [122]. In different PH models, not only NLRP3 complex was widely observed in the inflammatory cells around the pulmonary vessels [119, 123], but also NLRP3, ASC, and Caspase-1 proteins were abnormally upregulated in PAECs and PASMCs [124, 125]. Pulmonary vascular wall inflammation caused by various external stimuli (e.g., hypoxia, dust, and infection) is the basis of PH pathogenesis. The genetic defects in patients with PH (such as the loss of BMPR2 signaling) aggravate the activation of pyroptosis in pulmonary vascular cells [126]. These signals activate intracellular HIF-1α, NF-κB, caspase-8, and other pathways to promote the expression of NLRP3/caspase-1 [127, 128]. The effect of the non-classical regulatory pathway of pyroptosis in HPH has recently been identified for the first time [129]. Hypoxia increases caspase-4/caspase-11 expression in PAECs and further cleaves GSDMD. The former directly activates caspase-3 and mediates pyroptosis of PAECs and pulmonary vascular remodeling via the caspase-3/GSDME pathway.

### Pyroptosis and the treatment of PH

With the development of pyroptosis research, researchers have recognized the potential value of pyroptosis signaling in treating PH. The primary focus is on the regulation of the classical inflammasome pathway. For example, MnTE-2-PyP, a superoxide dismutase mimic, can reduce pulmonary vascular inflammation by scavenging oxygen free radicals in lung tissue, thereby inhibiting the levels of the NLRP3 inflammasome, caspase-1, IL-1β, and IL-18 from alleviating pulmonary vascular remodeling [130]. The κ-opioid receptor agonist U50448H provides another example, as it inhibits NLRP3/ caspase-1-induced pyroptosis in this case by activating cholinergic anti-inflammatory pathways and promoting the polarization of alveolar macrophages to the M2 type [131].

Recently, drugs targeting key signals in the classical pathways, particularly the NLRP3 inflammasome complex, have provided new options for treating PH. However, the therapeutic potential of NLRP3 protein inhibition in PH is still under debate. The NLRP3 inhibitor MCC950 interferes with the interaction between cardiomyocytes and macrophages in LPS-induced PH rats, maintains the balance of the immune microenvironment, and improves right ventricular dysfunction [132]. However, it has been observed that in NLRP3 knockout mice, the loss of NLRP3 expression is insufficient to inhibit caspase-1 expression under hypoxic conditions and hardly improves PH and RV remodeling [133]. Compared with NLRP3, inhibition of ASC or caspase-1 is a more promising molecular target for alleviating PH [120, 133]. Caspase-1 inhibitors (e.g., Ac-YVAD-CMK, VX-765) can alleviate pulmonary vascular remodeling, reduce right ventricular systolic pressure in MCT-induced PH rats, and significantly reduce the level of pulmonary fibrosis [134]. In addition, recent studies have reported that disulfiram targets GSDMD, the final effector of pyroptosis, and blocks the release of inflammatory factors by inhibiting the binding of N-GSDMD to acidic phospholipids on the cell membrane, thereby reversing the progression of PH [135]. However, these drugs have been validated in animal models, and their suitability for clinical use has yet to be assessed.

## Ferroptosis and cuproptosis

### Molecular regulation of ferroptosis

**Fig. 4:**
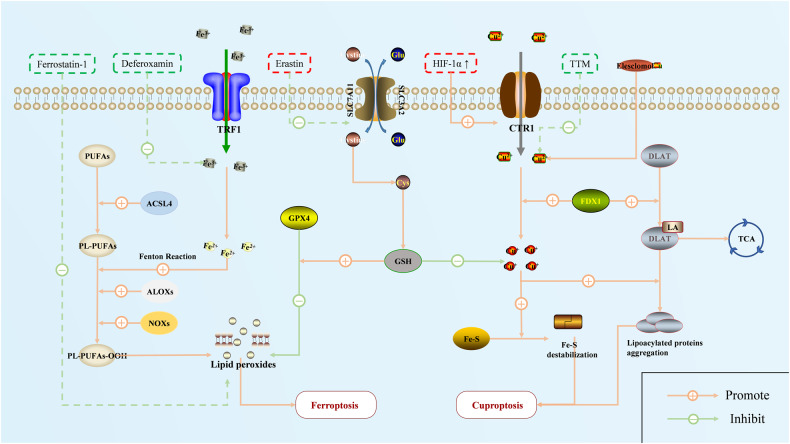
The processes and regulation of ferroptosis and cuproptosis. Although ferroptosis and cuproptosis have not been studied much in the context of PH, previous research has confirmed the feasibility of treating PH by interfering with these pathways. Red represents the enabler, and green represents the inhibitor.

Ferroptosis is an iron-dependent PCD process that has attracted significant attention recently. Although the exact mechanism of ferroptosis remains unknown, its development is inevitably accompanied by abnormal iron metabolism and the accumulation of lipid peroxides [136]. The cellular iron metabolism is regulated by the transport of transferrin and its receptor (TF/TFR1) on the cell membrane and the storage of intracellular ferritin [137, 138]. When cells are overloaded with iron, excess Fe^2+^ promotes the accumulation of lipid peroxidation and ROS through either enzymatic or non-enzymatic reactions (the Fenton reaction). Thereafter, ROS catalyzes the decomposition of polyunsaturated fatty acids (PUFAs) in the membrane phospholipid bilayer into aldehydes that are toxic to cells, leading to ferroptosis (Fig. 4) [139]. Because oxidative metabolism is mainly performed in mitochondria, the morphology of ferroptosis in cells usually manifests through rupture of the mitochondrial outer membrane, increased membrane density, and loss of ridge contraction. However, there is no obvious change in the nucleus, which differs from what is observed in other PCDs [140].

Ferroptosis is regulated by a combination of cellular sensitivity to lipid peroxidation and cellular defense against ferroptosis [141]. For example, an abnormal elevation of mitochondrial enzymes, lipoxygenase, and NADPH oxidase can promote lipid peroxidation, making cells susceptible to ferroptosis [141]. Glutathione peroxidase 4 (GPX4) is the classic core regulatory protein in ferroptosis and is mainly regulated by the Xc-GSH-GPX4 axis. This pathway transports cystine into the cell to mediate the synthesis of glutathione (GSH), which drives GPX4 to reduce lipid peroxides under the regulation of reduced GSH [142, 143]. In addition, the FSP1/CoQ10 axis, the GCH1/BH4 axis, and others can produce the corresponding antioxidant molecules (e.g., reduced CoQ10, BH4) to exert similar effects without GPX4 [144].

### Paradoxical ferroptosis states in PH

In PH, an imbalance in iron homeostasis is one of the mechanisms that worsens PH. However, existing studies have shown a few paradoxical phenomena. First, it has been established in PH models and clinical experiments that iron deficiency is strongly associated with poor outcomes in patients with PH, leading to inflammatory cell infiltration and right ventricular hypertrophy. Iron supplementation improves hemodynamic indices and prognostic outcomes [145–147]. However, the administration of ferroptosis inhibitors (deferoxamine or ferrostatin-1) reduces the production of lipid peroxide and inhibits pulmonary vascular remodeling in HPH rats [148]. Next, the anti-ferroptosis markers GPX4 and FTH1 are decreased, and the pro-ferroptosis signal NOX4 is increased in PAECs of HPH; however, GPX4 is increased in PASMCs, and both cell lines exhibit similar ferroptosis phenotypes, including ROS and MDA accumulation and decreased GSH levels [149, 150]. In addition, bioinformatics evidence has suggested the coexistence of the activation and silencing of ferroptosis in PH [151].

Studies on ferroptosis in PH are scarce, and the available conclusions are not always reliable. Since lipid peroxides are the ultimate effector signal for cell death and are upregulated in PH, we would prefer that ferroptosis is activated and contributes to it. On the one hand, it is believed that hypoxia can cause the activation of NOX enzymes, LOX-1, etc., and impair mitochondrial redox homeostasis in pulmonary vascular cells, which reduces the threshold for inducing ferroptosis (this death effect can occur at low iron levels) [152, 153]. On the other hand, although hypoxia can activate the SCL7A11/GPX4 pathway through HIF-1α and enhance cell defense, it is not sufficient to resist the high sensitivity of the cell to ferroptosis. This is also reflected in the fact that there is not enough GSH to provide sufficient redox substrates for elevated GPX4, which eventually causes further accumulation of lipid peroxides [154]. However, the specific mechanism by which ferroptosis aggravates PH is still unknown, and it may be related to ferroptosis products such as ROS and LPO. More fundamental research is needed to fill this gap.

### Cuproptosis and PH

As an essential micronutrient for the body, Cu homeostasis is widely involved in cellular activities such as energy metabolism, signal transduction, and biological macromolecular synthesis by regulating the activities of Cu-containing enzymes [155, 156]. Recently, Tsvetkov et al. demonstrated a novel ion-dependent PCD mode for the first time: cuproptosis [157]. When cells are overloaded with copper, ferredoxin 1 (FDX1) induces the synthesis of lipoacylase in the tricarboxylic acid cycle (TCA) and promotes the conversion of excess divalent copper ions to more toxic monovalent copper ions. Cu ions then promote the aggregation of lipoacylated proteins and trigger the destabilization of Fe-S cluster proteins, which leads to proteotoxic stress and cell death (Fig. 4) [157, 158], and this process cannot be reversed by other PCD inhibitors except for cuproptosis inhibitors such as copper chelators [157].

While there have been no studies on PH-related cuproptosis, an imbalance in copper homeostasis has long been observed in patients with PH. Clinical and basic studies have confirmed that the severity of PH is often accompanied by elevated copper ions in pulmonary vascular cells, possibly due to the upregulation of copper transporters induced by hypoxia or other factors [159, 160]. Because excess copper induces active cell death, cuproptosis may exacerbate PH progression, similar to pyroptosis or autophagy. A copper-chelating agent has been shown to induce a caspase-independent apoptosis pathway by activating AIF proteins to inhibit PAEC proliferation in patients with IPAH [161]. However, a copper-deficient diet fails to reverse pulmonary artery media thickening in HPH rats [161, 162], which may imply that cuproptosis is inhibited in PH and that high cellular copper levels result from passive elevation. In any case, the effect of cuproptosis on PH and whether cuproptosis targets are activated or inhibited in pulmonary vascular cells are promising topics that need investigation.

## Summary

The PCD between PASMCs and PAECs profoundly affects the progression of pulmonary hypertension. When cells face multiple pathogenic stimuli, apoptosis, autophagy, pyroptosis, ferroptosis, necroptosis, cuproptosis, or other PCD regulatory pathways that have not yet been discovered can collectively lead cells to the end of life. Regarding pulmonary vascular cells in the early stages of PH, the pro-death factors outweigh the anti-death factors. In the advanced stages of PH, a few PCDs, such as autophagy and pyroptosis, are still active. However, due to the inhibition of other PCDs, such as apoptosis, and the release of pro-proliferative signals during active cell death, the anti-death factors are stronger than the pro-death effects, which aggravates the proliferation of PASMCs and PAECs. Finally, this progression manifests as pulmonary artery thickening, muscularization, vascular remodeling, and right heart hypertrophy in lung tissues.

In drug development, although progress has been made in regulating pulmonary hypertension by modulating certain types of PCD pathways, most studies are only at the animal experimental stage and have not been validated for clinical trials, and it should be emphasized that when drugs are used to inhibit or promote one PCD to alleviate PH, the signaling crosstalk between PCD pathways may affect the regulation of other PCDs, which will directly determine the therapeutic effect and clinical outcome of the drug. Additionally, it is necessary to further understand the detailed mechanisms of PCD in PH and explore new modes of PCD in PH-related pulmonary vascular cells.
